# Supplementary material for: Unique molecular events during reprogramming of human somatic cells to induced pluripotent stem cells (iPSCs) at naïve state
Source: eLife. 2018 Jan 30;7:e29518. doi: 10.7554/eLife.29518 (PMC5807049; doi:10.7554/eLife.29518)
Supplement: Source data 1. — The Source Data files include the source R code for Figures 2–6. The R scripts use the data in the ‘Expression Data’ folder with the relative path. The ‘Expression Data’ folder contains the non-redundant related gene expression, methylation and histone modification tables for the main figures and figure supplements. [file elife-29518-data1.zip › Source Data/Expression data/Fig6A/naive.methylated.negative.gene.pvalue.pdf]

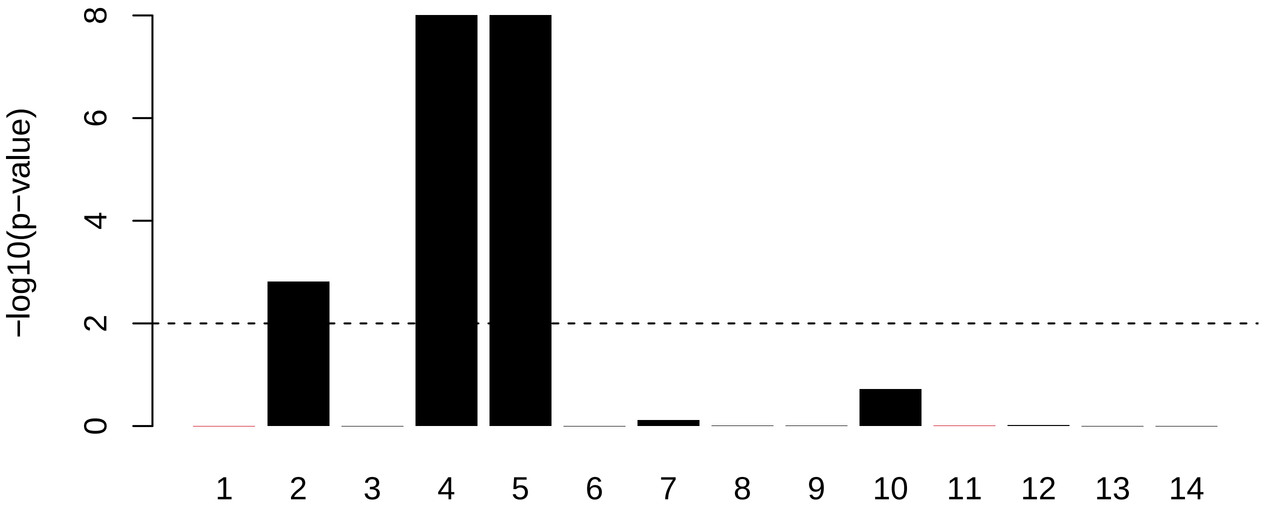
−log10(p−value)
0
2
4
6
8
1
2
3
4
5
6
7
8
9
10
11
12
13
14
