# Supplementary material for: Unique molecular events during reprogramming of human somatic cells to induced pluripotent stem cells (iPSCs) at naïve state
Source: eLife. 2018 Jan 30;7:e29518. doi: 10.7554/eLife.29518 (PMC5807049; doi:10.7554/eLife.29518)
Supplement: Transparent reporting form [file elife-29518-transrepform.docx]

***eLife’s* transparent reporting form**

We encourage authors to provide detailed information *within their submission* to facilitate the interpretation and replication of experiments. If you have any questions, please contact us: [editorial@elifesciences.org](mailto:editorial@elifesciences.org).

**Sample-size estimation**

- You should state whether an appropriate sample size was computed when the study was being designed
- You should state the statistical method of sample size computation and any required assumptions
- If no explicit power analysis was used, you should describe how you decided what sample (replicate) size (number) to use

Please outline where this information can be found within the submission (e.g., page numbers or figure legends), or explain why this information doesn’t apply to your submission:

We chose nine time-points during naïve reprograming to perform the integrative analysis of transcriptional and epigenetic dynamics in detail.

(P5 L7)

**Replicates**

- You should report how often each experiment was performed
- You should include a definition of biological versus technical replication
- The data obtained should be provided and sufficient information should be provided to indicate the number of independent biological and/or technical replicates
- If you encountered any outliers, you should describe how these were handled
- Criteria for exclusion/inclusion of data should be clearly stated
- High-throughput sequence data should be uploaded before submission, with a private link for reviewers provided (these are available from both GEO and ArrayExpress)

Please outline where this information can be found within the submission (e.g., page numbers or figure legends), or explain why this information doesn’t apply to your submission:

For RNA-seq experiments, we performed two biological replicates for samples at each time point, and three biological replicates for samples at 8d, 14d and 20d.

Samples with less than 1 million reads are excluded. All the sequencing derived data in this paper can be accessed in the GEO under GSE89072 (reviewer token: ebslgymgzjcbngt) fewfddd()swi()(<https://www.ncbi.nlm.nih.gov/geo/query/acc.cgi?acc=GSE89072>) with the secure token ebslgymgzjcbngt. (P23 L16)

**Statistical reporting**

- Statistical analysis methods should be described and justified
- Raw data should be presented in figures whenever informative to do so (typically when N per group is less than 10)
- For each experiment, you should identify the statistical tests used, exact values of N, definitions of center, methods of multiple test correction, and dispersion and precision measures (e.g., mean, median, SD, SEM, confidence intervals; and, for the major substantive results, a measure of effect size (e.g., Pearson's r, Cohen's d)
- Report exact p-values wherever possible alongside the summary statistics and 95% confidence intervals. These should be reported for all key questions and not only when the p-value is less than 0.05.

Fisher’s exact test was performed on the Figure 3-figure supplement 2A (P10 L22) and Figure 3-figure supplement1A (P8 L21). The detailed values of the test are listed in the table below.

| **TEST** | ***a*** | ***b*** | ***c*** | ***d*** |
| --- | --- | --- | --- | --- |
| Fisher’s exact test 1 (P10 L22) | 48 | 841 | 52 | 456 |
| Fisher’s exact test 2 (P8 L21) | 14 | 8315 | 191 | 1165 |
| Fisher’s exact test 3 (P8 L21) | 21 | 8386 | 184 | 1094 |
| Fisher’s exact test 4 (P8 L21) | 9 | 9033 | 196 | 447 |
| Fisher’s exact test 5 (P8 L21) | 20 | 8618 | 185 | 862 |
| Fisher’s exact test 6 (P8 L21) | 10 | 9086 | 195 | 394 |
| Fisher’s exact test 7 (P8 L21) | 23 | 8378 | 182 | 1102 |
| Fisher’s exact test 8 (P8 L21) | 40 | 8769 | 165 | 711 |

Please outline where this information can be found within the submission (e.g., page numbers or figure legends), or explain why this information doesn’t apply to your submission:

The symbols used in the table are represent the numbers described in the table below.

|  | Selected | Not selected |
| --- | --- | --- |
| In the specific cluster ( eg:8C-like genes) | a | c |
| Not in the specific cluster | d | b |

(For large datasets, or papers with a very large number of statistical tests, you may upload a single table file with tests, Ns, etc., with reference to page numbers in the manuscript.)

**Additional data files (“source data”)**

- We encourage you to upload relevant additional data files, such as numerical data that are represented as a graph in a figure, or as a summary table
- Where provided, these should be in the most useful format, and they can be uploaded as “Source data” files linked to a main figure or table
- Include model definition files including the full list of parameters used
- Include code used for data analysis (e.g., R, MatLab)
- Avoid stating that data files are “available upon request”

Please indicate the figures or tables for which source data files have been provided:

The R code files for each plot are included as source data.
